# Enhancement of ATP generation capacity, antioxidant activity and immunomodulatory activities by Chinese Yang and Yin tonifying herbs

**DOI:** 10.1186/1749-8546-2-3

**Published:** 2007-03-27

**Authors:** Kam Ming Ko, Hoi Yan Leung

**Affiliations:** 1 Department of Biochemistry, Hong Kong University of Science and Technology, Clear Water Bay, Hong Kong, China

## Abstract

Chinese tonifying herbs such as *Herba Cistanche*, *Ganoderma *and *Cordyceps*, which possess antioxidant and/or immunomodulatory activities, can be useful in the prevention and treatment of age-related diseases. Pharmacological studies on Yang and Yin tonifying herbs suggest that Yang tonifying herbs stimulate mitochondrial adenosine triphosphate (ATP) generation, presumably through the intermediacy of reactive oxidant species, leading to the enhancement of cellular/mitochondrial antioxidant status. Yin tonifying herbs, however, apart from possessing antioxidant properties, exert mainly immunomodulatory functions that may boost a weak immune system and may also suppress overreactive immune responses. The abilities of Yang and Yin Chinese tonifying herbs to enhance ATP generation and to exhibit antioxidant and/or immunomodulatory actions are the pharmacological basis for their beneficial effects on the retardation of aging.

## Background

Aging is a process of bodily change with time, leading to increased susceptibility to disease, and ultimately death. Because reactive oxidant species (ROS) and immune dysfunction are major causes of age-related diseases [1-3], the maintenance of antioxidant and immune fitness is a rational approach to preventive health care. Accumulation of ROS-induced oxidative damage to DNA, proteins, and other macromolecules has been regarded as a major endogenous cause of aging [1]. In addition to ROS-mediated cellular damage, aging was found to be associated with immune senescence, attributable at least partly to the loss of T lymphocyte functions [2,3]. Such loss increases the prevalence of infectious diseases in the elderly. With advances in modern medical research techniques, research on age-related chronic illnesses has become intense, in the quest for valuable preventive and therapeutic measures. Humans have been making continuous efforts to fight aging. As Chinese medicine has always emphasized the prolongation of a healthy lifespan, many Chinese tonifying herbs have long been used to safeguard health and to delay the onset of senility.

Under both normal and pathological conditions, ROS are generated in all cells undergoing aerobic metabolism, particularly from mitochondria. The cell possesses two distinct antioxidant defense systems to counteract damaging ROS: (1) enzymatic antioxidants such as catalase, superoxide dismutase (SOD), glutathione peroxidase and other related enzymes/molecules, and (2) non-enzymatic antioxidants such as ascorbic acid (vitamin C), α-tocopherol (vitamin E) and β-carotene. To achieve optimal antioxidant fitness, every component of the antioxidant defense system should function optimally because antioxidants must work together in a synergistic manner. Chinese tonifying herbs have been shown to possess both *in vitro *and *in vivo *antioxidant activities [4,5].

The immune system fights against 'foreign invaders' such as bacteria, viruses, fungi, yeasts and parasites. The humoral and cell-mediated immune responses show great competence in dealing with intruders. Moreover, the surveillance function of the immune system tends to prevent cancers, particularly in old age. However, an overreactive or imbalanced immune system can cause allergies or autoimmune disorders. A well-constituted and balanced immune system is thus crucial for safeguarding health. Chinese tonifying herbs have been shown to stimulate or suppress the cell-mediated immune response both *in vitro *and *in vivo *[6].

The importance of disease prevention has been recognized by Chinese medicine through experience accumulated over centuries. Many Chinese tonifying herbs have long been used for safeguarding health and for delaying the onset of senility. According to Chinese medicine theories, tonifying herbs prescribed for various symptoms of ill-health are generally classified into four categories on the basis of their health-promoting actions, namely 'Yang-invigorating', the '*Qi*-invigorating', the 'Yin-nourishing' and the 'Blood-enriching' herbs [7]. The '*Qi*-invigorating' and 'Blood-enriching' herbs are of Yang and Yin characteristics respectively. Chinese medicine theories suggest that a balance of Yin and Yang is essential to sustain optimal body function [8]. From a modern medical perspective, the maintenance of Yin and Yang in harmony may be described as the attainment of bodily homeostasis. The long-known antagonistic relationship between parasympathetic and sympathetic neural activities affords an example of both a phenomenon well-recognized by Western medicine and the Yin/Yang balance. A recent psychophysiological investigation in humans revealed an association between decreased parasympathetic or sympathetic activities with deficiencies of Yin or Yang respectively [9].

The theoretical framework of Chinese medicine is based on the Chinese cultural fabrics and clinical experience, while modern Western medicine has been established on the basis of laboratory and clinical investigations [10]. As the two distinct medical systems are complementary, bridging of the knowledge gap between Chinese and Western medicine is essential for their integration, in clinical practice, for disease prevention and treatment. Expounding Chinese medicinal theories in modern scientific terms to a Western audience facilitates communication between practitioners of the two systems.

In our earlier studies, we found that tonifying herbs with Yang or Yin properties were associated with antioxidant and immunostimulatory activities respectively [4]. Recent studies indicated that only Yang tonifying herbs (not Yin tonifying herbs) enhanced mitochondrial ATP generation capacity in mouse hearts [11]. We therefore suggest that Yang tonifying herbs enhance mitochondrial ATP generation, while Yin tonifying herbs are associated with immunomodulatory activities. In this mini-review, we summarize the abilities of Yang and Yin tonifying herbs to enhance ATP generation capacity, and to potentiate antioxidant and/or immunomodulatory actions, in an effort to characterize their respective pharmacological properties.

### Enhancement of ATP generation by Yang tonifying herbs

In Chinese medicinal theories, Yang is a manifestation of body functions supported by various organs. A 'Yang-invigorating' action therefore involves the enhancement of bodily functions in general and cellular activities that consume ATP in particular. The mitochondrion is responsible for the generation of ATP through oxidative metabolism. To establish the pharmacological basis of 'Yang-invigorating' action, we have recently investigated the effect of Yang herbs on ATP generation capacity in heart homogenates prepared from mice that were pretreated with methanolic extracts of herbs [11]. Tonifying herbs from other functional categories were examined for comparison. While Chinese herbs are usually extracted by water for human oral consumption, water was replaced by methanol in our study for convenience in the processing and storage of samples. Yang herbs invariably enhanced myocardial ATP generation, with stimulation ranging from 20–130%. *Herba Cynomorii *and *Semen Cuscutae *were the most potent herbs examined. By contrast, none of the Yin herbs enhanced ATP generation; some Yin herbs even suppressed ATP generation slightly (Table 1). A preliminary mechanistic study indicated that Yang herbs may speed up ATP synthesis by increasing mitochondrial electron transport [11].

**Table 1:** Effect of Yang and Yin tonifying herbs on myocardial ATP generation capacity in mice *ex vivo*

Herbs	ATP generation (% control)	Herbs	ATP generation (% control)
Yang herbs		Yin herbs	
*Cortex Eucommiae*	157 ± 11.2 *	*Semen Prinsepiae*	95.5 ± 12.1
*Herba Cistanches*	191 ± 3.20 **	*Fructus Ligustri*	101 ± 5.00
*Herba Cynomorii*	230 ± 17.3 **	*Rhizoma Polygonati*	102 ± 4.53
*Rhizoma Curculiginis*	149 ± 38.0	*Radix Asparagi*	98.2 ± 9.92
*Herba Epimedii*	130 ± 7.51 *	*Radix Ophiopogonii*	102 ± 8.51
*Radix Dipsaci*	120 ± 19.7	*Radix Oryzae*	85.0 ± 7.43
*Rhizoma Drynariae*	154 ± 14.6 *	*Herba Dendrobii*	82.7 ± 0.92 *
*Fructus Psoraleae*	175 ± 13.4 *	*Herba Ecliptae*	80.3 ± 7.05 *
*Semen Cuscutae*	222 ± 2.12 **		
*Radix Morindae*	142 ± 19.3		
*Semen Allii*	133 ± 35.8		

Mice were pretreated with herbal extracts at daily doses of 1 g/kg for 3 days. The mean value of myocardial ATP generation capacity in unpretreated mice was 147 ± 17.6 (S.D.) nmol ATP/mg protein/10 min, (n = 6). * *P *< 0.05; ** *P *< 0.01, Student's t test

### Correlation between enhancement of ATP generation capacity and antioxidative capacity

Mitochondrial oxidative phosphorylation generates ROS as byproducts. Highly reactive chemically, ROS attack cellular structures located near the sites where ROS are generated. Mitochondrial DNA, proteins, and lipids in the inner membrane of mitochondria are thus vulnerable to oxidative damage [12], resulting in generalized organelle dysfunction, defective mitochondrial biosynthesis and poor energy metabolism [13].

Under normal physiological conditions, the mitochondrial antioxidant defense system adequately handles the potentially detrimental effects of ROS derived from energy metabolism [14]. When a functional imbalance between ROS levels and antioxidant concentrations caused by various disease states and/or aging occurs, age-related disorders such as cancer, cardiovascular diseases, brain dysfunction, or cataract may occur [15]. Antioxidant supplementation, particularly from herbal extracts, has become a trend in preventive health care.

Using an oxygen radical absorbance capacity assay, Ou *et al*. recently compared the free radical scavenging (i.e. antioxidant) activities of Yang and Yin herbs [16]. The results indicated that Yin herbs generally possessed higher antioxidant activities than Yang herbs and that the antioxidant potencies correlated well with the amounts of total phenolic compounds in the herbs. The authors suggested an analogy between Yin/Yang balance and antioxidation/oxidation in energy metabolism. These findings of higher antioxidant activities in Yin herbs as compared with those in Yang herbs do not agree with the findings from one of our earlier studies which showed that most of the Yang herbs possessed a more potent 1,1-diphenylpicryhydrazyl radical-scavenging action than other tonifying herbs [4] (Table 2). Although the use of different herbal extraction methods and distinct antioxidant assays precludes direct comparison of the two studies, the discrepancy might be due to the selection of almost completely different sets of Yin and Yang herbs for testing in the two studies. Our study focused on herbs used for safeguarding health (i.e. herbs used for tonifying purposes) (Tables 2, Table 3 of reference [17]). Ou *et al*. probably used a selection criterion based on the general Yin and Yang properties of the herbs instead of their Yin-tonifying and Yang-tonifying actions [16]. Szeto and Benzie, using the same set of herbs described in Ou *et al*. to examine possible protective effects on DNA oxidative damage, found that the Yang herbs showed an antioxidant effect superior to that of Yin herbs [5].

**Table 2:** Antioxidant and immunomodulatory activities of Chinese tonifying herbs

	^a^DPPH radical scavenging IC50 (mg/ml)	^b^Immunomodulatory index *in vitro*	^c^Immunomodulatory index *ex vivo*
Control		1.00 ± 0.03	1.00 ± 0.05
Yang herbs			
*Cortex Eucommiae*	> 5	0.46 ± 0.02*	1.04 ± 0.09
*Fructus Psoraleae*	1.0 ± 0.0	1.42 ± 0.02*	1.23 ± 0.01*
*Herba Cistanches*	1.8 ± 0.0	0.75 ± 0.13	0.95 ± 0.12
*Herba Epimedii*	1.1 ± 0.1	0.57 ± 0.03*	0.98 ± 0.01
*Radix Dipsaci*	0.8 ± 0.0	0.42 ± 0.08*	1.02 ± 0.02
*Radix Morindae*	> 5	2.16 ± 0.10*	1.31 ± 0.04*
*Rhizoma Cibotii*	0.6 ± 0.0	0.16 ± 0.03*	0.97 ± 0.04
*Rhizoma Drynariae*	> 5	0.59 ± 0.09*	0.98 ± 0.08
Yin herbs			
*Fructus Ligustri*	0.5 ± 0.0	1.73 ± 0.07*	1.80 ± 0.17*
*Herba Dendrobii*	1.4 ± 0.1	2.54 ± 0.09*	1.59 ± 0.09*
*Herba Ecliptae*	> 5	1.65 ± 0.02*	1.27 ± 0.12*
*Radix Asparagi*	> 5	0.70 ± 0.02*	1.24 ± 0.05*
*Radix Ophiopogonis*	> 5	1.65 ± 0.05*	1.44 ± 0.11*
*Radix Oryzae*	> 5	0.78 ± 0.13	0.97 ± 0.04
*Rhizoma Polygonati*	3.5 ± 0.3	1.43 ± 0.09*	1.21 ± 0.06*
*Semen Prinsepiae*	> 5	1.70 ± 0.03*	1.39 ± 0.11*

^a^Methanol extracts of tonifying herbs were subjected to the DPPH assay. Values given are means ± S.D., (n = 3). (DPPH scavenging activity was regarded as negligible if the IC_50 _was > 5 mg/ml).

^b^Splenocytes isolated from mice were cultured in 96-well microtiter plates in a final volume of 100 μl of culture medium, with the respective methanol extracts added at final concentrations ranging from 15.6–1000 μg/ml. Values given are means ± S.E.M., (n = 4).

^c^Animals were pretreated orally with the methanol extracts at a daily dose of 1 g/kg for 3 days. All animals were sacrificed 24 hours post-dosing. Splenocytes isolated from pretreated animals were cultured in microtiter plates in a final volume of 100 μl culture medium. Values given are means ± S.E.M., (n = 3–5).

* Significantly different from the control group (*P *< 0.05)

**Table 3:** Nomenclature and classification of selected Yang and Yin tonifying herbs *

Pharmaceutical name (Chinese *pinyin*)	Plant part used	Latin botanical name
**Yang tonifying herbs**		
*Cortex Eucommiae *(*Duzhong*)	Bark	*Eucommia ulmoides *Oliv.
*Fructus Psoraleae *(*Buguzhi*)	Fruit	*Psoralea corylifolia *L.
*Herba Cistanches *(*Roucongrong*)	Whole plant	*Cistanche salsa *(C.A. Meyer) G. Beck.
*Herba Epimedii *(*Yinyanghuo*)	Whole plant	*Epimedium grandiflorum *Morr.
*Radix Dipsaci *(*Xuduan*)	Root	*Dipsacus japonicus *Miq.
*Radix Morindae *(*Bajitian*)	Root	*Morinda officinalis *How
*Rhizoma Cibotii *(*Gouji*)	Rhizome	*Cibotium barometz *(L.) J. Sm.
*Rhizoma Drynariae *(*Gusuibu*)	Rhizome	*Drynaria fortunei *(Kunze) J. Sm.
*Semen Cuscutae *(*Tusizi*)	Seed	*Cuscuta chinensis *Lam.
*Herba Cynomorii *(*Suoyang*)	Whole plant	*Cynomorium songricum *Rupr.
**Yin tonifying herbs**		
*Fructus Ligustri *(*Nuzhenzi*)	Fruit	*Ligustrum lucidum *Ait.
*Herba Dendrobii *(*Shihu*)	Whole plant	*Dendrobium nobile *Lindl.
*Herba Ecliptae *(*Mohanlian*)	Whole plant	*Ecliptae prostrata *L.
*Radix Asparagi *(*Tianmendong*)	Root	*Asparagus cochinchinensis *(Lour.) Merr.
*Radix Ophiopogonis *(*Maimendong*)	Root	*Ophiopogon japonicus *(L. f.) Ker-Gawl.
*Radix Oryzae *(*Nuodaogenxu*)	Root	*Oryza sativa *L.
*Rhizoma Polygonati *(*Yuzhu*)	Rhizome	*Polygonatum odoratum *(Mill.) Druce
*Semen Prinsepiae *(*Ruiren*)	Seed	*Prinsepia uniflora *Batal.
*Semen Sesami *(*Heizhima*)	Seed	*Sesamum indicum *L.
**'*Fu Zheng*' herb**		
*Ganoderma *(*Lingzhi*)	Fruiting body	*Ganoderma lucidum *(Leyss. Ex Fr.) Karst
**Yin-Yang tonifying herb**		
*Cordyceps *(*Dongchongxiacao*)	Whole plant	*Cordycep sinensis*

*Adapted from Liang [17]

### Antioxidant activities of Yang tonifying herbs

Several Yang herbs have been shown to possess antioxidant activities both *in vitro *and *in vivo *(Table 4). *In vitro *free radical-scavenging activities were detected in herbal extracts prepared from *Herba Epimedii *[4,18], *Radix Dipsaci *[4,16], *Fructus Psoraleae *[4], *Semen Cuscutae *[16], *Herba Cistanche *[4,16,18], *Cortex Eucommiae *[19] and *Rhizoma Cibotii *[4,16]. Aqueous extracts of *Rhizoma Drynariae *and *Cortex Eucommiae *were found to inhibit oxidant production from rat osteoblasts [20], and also inhibited biomolecular oxidative damage [21]. Active ingredients (bakuchiol, isobavachin and isobavachalcone) from *Fructus Psoraleae *inhibited the NADPH-dependent peroxidation of rat microsomal and mitochondrial lipids *in vitro *[22]. An ethanolic extract of *Radix Dipsaci *enhanced the antioxidant status of blood and liver in rodents [23] and a *Radix Morindae *extract increased blood antioxidant enzyme activities in diabetic rats [24]. Phenylethanoids isolated from *Herba Cistanche *were found to prevent cell damage induced by *in vitro *and *in vivo *exposure to carbon tetrachloride in rats [25]. A recent study from our laboratory indicated that pretreatment with the methanolic extract of *Herba Cistanche *protected against ischemia-reperfusion injury in rat hearts *ex vivo *and enhanced mitochondrial ATP generation in the rat hearts *ex vivo *and H9c2 cells *in situ*. The ATP-stimulating action was possibly due to enhanced oxidative phosphorylation caused by increases in the activities of complexes I and III [26]. As good body function requires a large amount of energy and antioxidant defense is essential in sustaining mitochondrial ATP production [27], the antioxidant activities of Yang herbs may safeguard ATP generation, particularly under conditions of upregulated cellular activities.

**Table 4:** Antioxidant activities of Yang tonifying herbs

HERBS	ANTIOXIDANT ACTIVITIES	REFS
*Herba Epimedii*	water extract caused development of superoxide scavenging activity	[18]
*Rhizoma Drynariae*	water extract decreased oxidant production in rat osteoblasts water extract caused development of inhibitory effect on biomolecular oxidative damage	[20] [21]
*Radix Dipsaci*	ethanol extract enhanced blood and liver antioxidant status in rats and mice	[23]
*Fructus Psoraleae*	active ingredients (bakuchiol, isobavachin, isobavachalcone) inhibited the NADPH-dependent peroxidation of liver microsomal and mitochondrial lipids *in vitro *in rats	[22]
*Semen Cuscutae*	acetone/water (1:1, v/v) extract caused development of oxygen radical scavenging activity	[16]
*Herba Cistanche*	water extract caused development of superoxide scavenging activity phenylethanoids prevented cell damage induced by exposure to carbon tetrachloride *in vitro *and *in vivo*	[18] [25]
*Cortex Eucommiae*	water extract caused development of hydroxyl radical scavenging activity water extract caused development of inhibitory effect on biomolecular oxidative damage	[19] [21]
*Radix Morindae*	ethanol extract increased blood superoxide dismutase and catalase activities in diabetic rats	[24]
*Rhizoma Cibotii*	acetone/water (1:1, v/v) extract caused development of oxygen radical scavenging activity	[16]

### Antioxidant activities of Yin tonifying herbs

Methanolic extracts of both *Fructus Ligustri *and *Herba Ecliptae *were found to enhance hepatic glutathione (GSH) regeneration capacity in rats [4,28]. The enhancement of hepatic GSH regeneration capacity by *Fructus Ligustri *was associated with a hepatoprotective action against carbon tetrachloride toxicity [28]. Activity-directed fractionation of *Fructus Ligustri *indicated that the hepatoprotective principle(s) resided mainly in the oleanolic acid-enriched butanol and chloroform fractions [28]. Moreover, our recent studies showed that both short and long term pretreatment with oleanolic acid protected against myocardial ischemia-reperfusion injury in rats [29,30]. It was suggested that the cardioprotection afforded by oleanolic acid pretreatment was related to the enhancement of mitochondrial antioxidant mechanism mediated by GSH and α-tocopherol [29]. Both experimental and clinical investigations indicated that the antioxidant status influenced immunocompetence, particularly under conditions of stress such as physical exercises or chronic diseases [31]. The antioxidant activities of Yin tonifying herbs may positively influence immunostimulatory activities.

### Experimental studies on a 'Yang-invigorating' herbal formula

A 'Yang-invigorating' herbal formula named VI-28 has been shown to produce 'Yang-invigorating' effects [32] and enhance red cell antioxidant status, particularly Cu-Zn-superoxide dismutase (SOD) activity, in elderly male human subjects [33]. This herbal formula is comprised of *Radix Ginseng*, *Cornu Cervi*, *Cordyceps*, *Semen Allii*, *Fructus Cnidii*, *Fructus Evodiae *and *Rhizoma Laemferiae*. Recently we investigated the effects of long-term VI-28 treatment on red cell Cu-Zn-SOD activity, mitochondrial functional ability, and antioxidant levels, in various tissues of rats of both sexes [34]. The results indicated that VI-28 treatment increased red cell Cu-Zn-SOD activity and mitochondrial ATP generation capacity, increased the levels of reduced GSH and α-tocopherol, and reduced Mn-SOD activities. The enhancement of ATP generation by VI-28 increased mitochondrial ROS production, resulting in the upregulation of mitochondrial antioxidant mechanism. The VI-28-induced increase in mitochondrial antioxidant capacity in various tissues was evidenced by a significant reduction in ROS generation. Given that cellular energy status and mitochondrial ROS generation are factors critically involved in aging, the dual effect of 'Yang-invigoration' produced by VI-28 may have clinical implications in the prevention of age-related diseases.

### Immunomodulatory activities of Yin tonifying herbs

It was suggested that the proper functioning of the immune system requires dynamic interactions between Yang and Yin. And while the antigen-nonspecific immune response is associated with Yang, the antigen-specific response is related toYin [35]. One of our earlier studies investigated antioxidant and immunomodulatory activities in different categories of tonifying herbs. The results showed that 6 and 7 of a total of 8 Yin herbs tested potentiated concanavalin A (Con A)-stimulated splenocyte proliferation (an antigen-specific response) in mice *in vitro *and *ex vivo *respectively. By contrast, only 3 of 9 Yang herbs tested showed a similar enhancement of the Con A-stimulated immune response [4] (Table 2).

Among the Yin herbs, the methanolic extract of *Fructus Ligustri *yielded the most robust immunostimulatory action in mouse splenocytes [4]. Differential extraction of *Fructus Ligustri *by solvents of increasing polarity indicated that the immunostimulatory activity resided mainly in the petroleum ether fraction [36]. Oleanolic acid, an immunomodulatory triterpenoid commonly found in herbs including *Fructus ligustri *[37,38], was undetectable in this fraction [36]. Currently, activity-directed fractionation of the petroleum ether extract of *Fructus Ligustri *is under way in our laboratory. Various immunomodulatory actions of Yin tonifying herbs, and the active ingredients of the herbs, have been reported in other studies (Table 5). An aqueous extract of *Radix Asparagi *was found to inhibit tissue necrosis factor-α (TNF-α) secretion by suppressing Interleukin (IL)-2 secretion from astrocytes, implicating that the extract might exhibit anti-inflammatory activity in the central nervous system [39]. Both the crude aqueous extract and the two active ingredients (ruscogenin and ophiopogonin D) of *Radix Ophiopogonis *produced anti-inflammatory effects in rodents [40]. While the aqueous extract inhibited xylene-induced ear swelling and carrageenan-induced paw edema in mice, it also suppressed carrageenan-induced pleural leukocyte migration in rats, and the zymosan-evoked migration of peritoneal total leukocytes and neutrophils in mice. Treatments with ruscogenin and ophiopogonin D decreased zymosan-induced peritoneal leukocyte migration in mice and reduced the phorbol-12-myristate-13 acetate-induced adhesion of HL60 cells to ECV304 cells [40]. Several sesquiterpenes isolated from *Herba Dendrobii *were found to exhibit immunomodulatory activity by exerting comitogenic effects on Con A and lipopolysaccharide-stimulated mouse splenocytes [41,42]. It has recently been reported that an ethanolic extract of black rice (the fruit of *Oryza sativa*) showed anti-asthmatic effects in a mouse model [43]. Treatment with the ethanolic extract of black rice reduced the number of eosinophils in bronchoalveolar lavage fluid, alleviated the airway hyper-response, and decreased the extent of airway inflammation in ovalbumin (OVA)-immunized and -aerolized mice challenged with OVA. Moreover, the ethanolic extract treatment decreased interferon-γ (INF-γ), IL-4, IL-5 and IL-13 levels in the supernatants of cultured splenocytes and suppressed the plasma levels of OVA-specific immunoglobulin (Ig)G, IgG2α, IgG1 and total IgE in OVA-immunized and -challenged mice [43]. Clinical investigations indicated that intramuscular injection of undiluted *Fructus Ligustri *extract at a dose of 2–4 ml once or twice daily could prevent leucopenia caused by chemotherapy or radiotherapy. *Fructus Ligustri *treatment normalized white blood cell counts, thereby increasing tolerance to chemo/radiotherapy [44]. Oral administration of *Fructus Ligustri *tablets at a daily dose of 50 g equivalence of crude herb was found to ameliorate the symptoms of chronic bronchitis [44]. A herbal formula comprising *Fructus Ligustri*, *Radix Scutellariae*, *Radix Astragalus *and *Eupolyphaga et polyphae *was found to alleviate symptoms and improve immune function in HIV/AIDS patients [45].

**Table 5:** Immunomodulatory activities of Yin tonifying herbs

HERBS	IMMUNOMODULATORY ACTIVITIES	REFS
*Fructus Ligustri*	Methanolic extract or petroleum ether fraction enhanced Con A-stimulated proliferation of mouse splenocytes *in vitro *and *ex vivo*	[4,36]
*Radix Asparagi*	Water extract inhibited TNF-α secretion by suppressing IL-2 secretion from astrocytes	[39]
*Radix Ophiopogonis*	Water extract inhibited xylene-induced ear swelling and carrageenan-induced paw edema in mice Active ingredients (ruscogenin and ophiopogonin D) decreased zymosan-induced adhesion of HL60 cells to ECV304 cells	[40]
*Herba Dendrobii*	Active ingredients (sesquiterpenes) showed a co-mitogenic effect on Con A and lipopolysaccharide-stimulated mouse splenocytes	[41,42]
*Radix Oryza*	Ethanolic extract of black rice (the fruit of *Oryza sativa*) decreased the extents of airway inflammation and hyper-response in OVA-immunized and aerolized OVA-challenged mice Ethanolic extract of black rice decreased various cytokine levels in the supernatant of cultured splenocytes and suppressed the plasma levels of OVA-specific IgG and total IgE in OVA-immunized and challenged mice	[43]

### Ganoderma – A '*Fu Zheng*' tonifying herb

*Ganoderma*, another Yin tonifying herb with immunomodulatory effects, is widely consumed by the Chinese people who believe that it promotes health and longevity, lowers the risk of cancer and heart diseases and boosts the immune system [46]. In Chinese medicine, *Ganoderma *is regarded as a very potent herb for '*Fu Zheng*', a Chinese medicine concept comparable to immunotherapy/immunomodulation in Western medicine. While *Ganoderma *is traditionally used to increase the resistance of the body immune system to pathogens and to restore normal body functions, the herb has now also been used to decrease the side effects of Western medical procedures, such as surgery, radiotherapy and chemotherapy which often weaken the immune system. The anti-cancer/immunomodulatory effects of *Ganoderma *were associated with triterpenes [47], polysaccharides [48,49] or immunomodulatory proteins [50] through mechanisms involving inhibition of DNA polymerase [51], inhibition of post-translational modification of the Ras oncoprotein [52] or the stimulation of cytokine production [53]. Recent studies on the immunomodulatory activities of *Ganoderma *indicated that *Ganoderma *extract stimulated the proliferation of human peripheral blood mononuclear cells and raised the levels of mRNAs encoding Th1 and Th2 cytokines in these cells [54]. Moreover, polysaccharides of *Ganoderma *activated mouse splenic B cells and induced these cells to differentiate into IgM-secreting plasma cells. This process was dependent on the polysaccharide-mediated induction of Blimp-1, a master regulator capable of triggering a cascade of gene expression during plasmacytic differentiation [55]. In human peripheral B lymphocytes, the *Ganoderma *polysaccharide fraction enhanced antibody secretion and induced the production of Blimp-1 mRNA, though it failed to induce lymphocyte differentiation [55].

In addition to immunomodulating activities, *Ganoderma *possesses *in vivo *antioxidant potential, another aspect of Yin tonifying action. Treatment with *Ganoderma *extract was found to enhance the hydroxyl radical scavenging activity of rabbit blood plasma [56,57]. *Ganoderma *acted by stimulating cellular and mitochondrial SOD activities, thereby enhancing the antioxidant capacity of the body [58]. It was shown that an intraperitoneal injection of *Ganoderma *extract following a lethal dose of cobalt X-ray radiation caused a marked prolongation of survival time in mice [59]. Pretreatment with *Ganoderma *extract also markedly protected against carbon tetrachloride-induced hepatic damage and the associated impairment in hepatic antioxidant status [60].

### Cordyceps – A Yin/Yang tonifying herb

*Cordyceps*, a premium Chinese tonifying herb which replenishes the 'kidney' and soothes the 'lung', is prescribed for the treatment of a host of disorders, including hyposexualities, hyperglycemia, hyperlipidemia, asthenia after illness, respiratory diseases, renal disorders, liver and heart diseases [61]. *Cordyceps *is regarded as a tonifying herb with both 'Yin-nourishing' and 'Yang-invigorating' actions. Pharmacological studies have shown that *Cordyceps *possesses a wide spectrum of biological activities including antioxidation [61-64], immunopotentiation [65-68], anti-tumorigenesis [68-71], anti-inflammation [72] and stimulation of testosterone biosynthesis [73]. We have recently investigated the effects of wild and cultured *Cordyceps *on Con A-stimulated splenocytes (an *in vitro *bioassay for Yin tonifying action) and myocardial ATP generation capacity (an *ex vivo *bioassay for Yang tonifying action) [74]. The results indicated that methanolic extracts of wild and cultured *Cordyceps *enhanced both the Con A-stimulated splenocyte proliferation *in vitro *and myocardial mitochondrial ATP generation *ex vivo *in mice, with no significant difference in potencies when the two types of *Cordyceps *were compared. While the immunopotentiating effect was associated with an increase in IL2 production, the stimulation of myocardial ATP generation was paralleled by an enhancement in mitochondrial electron transport. When compared with typical Yin and Yang tonifying herbs (*Fructus Ligustri *and *Herba Cynomorii *respectively), *Cordyceps *was found to possess both Yin and Yang tonifying actions, with a lower potency in both modes of action. The observation of both immunopotentiating and ATP-enhancing activities in *Cordyceps *extracts further supports the pharmacological basis of Yin and Yang tonifying herbs in Chinese medicine.

## Conclusion

**Figure 1:**
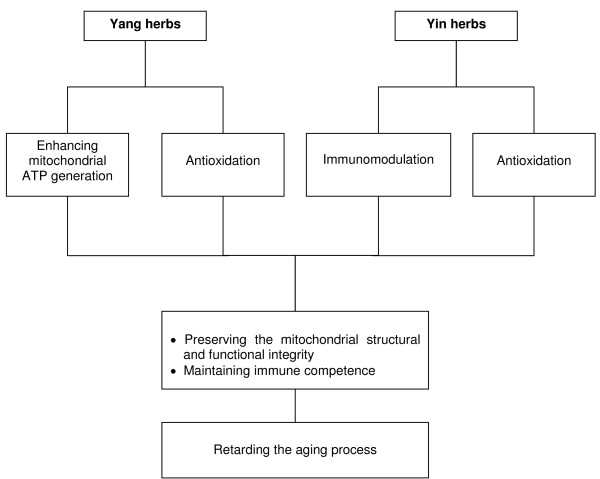
Anti-aging potential of Chinese tonifying herbs.

Yang tonifying herbs stimulate mitochondrial ATP generation, leading to the enhancement of cellular/mitochondrial antioxidant status, presumably through the intermediacy of ROS. Yin tonifying herbs, which also possess antioxidant properties, are mainly immunomodulatory, thereby boosting weak immune functions and suppressing overreactive or unbalanced immune responses. *Cordyceps*, highly regarded as a tonifying herb with a dual action of Yin and Yang, stimulates mitochondrial ATP generation and enhances cellular immune responses. Given that impairment in mitochondrial functional ability and antioxidant status, and a decline in immunocompetence, are believed to be critically involved in the development of age-related diseases and the aging process, the abilities of Yang and Yin tonifying herbs to enhance ATP generation capacity and to produce antioxidant and immunomodulatory actions are beneficial for safeguarding health and delaying the onset of senility (Figure 1). While animal models may be used for testing working hypotheses on Yang and Yin tonifying actions, clinical studies, using Yang and Yin tonifying herbs and/or defined chemicals isolated from the herbs or synthesized in the laboratory, on age-related variations in antioxidant and immune function, would be of considerable value.

## List of abbreviations

ATP: adenosine triphosphate

Con A: concanavalin A

GSH: reduced glutathione

Ig: immunoglobulin

IL: interleukin

INF: interferon

OVA: ovalbumin

ROS: reactive oxygen species

SOD: superoxide dismutase

TNF: tissue necrosis factor

## Competing interests

The author(s) declare that they have no competing interests.

## Authors' contributions

KMK conceived and wrote the article. HYL did literature research and organized the information.
